# Novel Antineuronal Autoantibodies With Somatodendritic Staining Pattern in a Patient With Autoimmune Psychosis

**DOI:** 10.3389/fpsyt.2020.00627

**Published:** 2020-08-06

**Authors:** Dominique Endres, Sebastian Rauer, Alexander Pschibul, Patrick Süß, Nils Venhoff, Kimon Runge, Bernd Feige, Dominik Denzel, Kathrin Nickel, Tina Schweizer, Simon Maier, Karl Egger, Katharina Domschke, Philipp T. Meyer, Harald Prüss, Ludger Tebartz van Elst

**Affiliations:** ^1^ Section for Experimental Neuropsychiatry, Department of Psychiatry and Psychotherapy, Medical Center - University of Freiburg, Faculty of Medicine, University of Freiburg, Freiburg, Germany; ^2^ Department of Psychiatry and Psychotherapy, Medical Center - University of Freiburg, Faculty of Medicine, University of Freiburg, Freiburg, Germany; ^3^ Department of Neurology, Medical Center - University of Freiburg, Faculty of Medicine, University of Freiburg, Freiburg, Germany; ^4^ Department of Neuropediatrics and Muscle Disorders, Medical Center - University of Freiburg, Faculty of Medicine, University of Freiburg, Freiburg, Germany; ^5^ Department of Molecular Neurology, University Hospital Erlangen, Erlangen, Germany; ^6^ Department of Rheumatology and Clinical Immunology, Medical Center - University of Freiburg, Faculty of Medicine, University of Freiburg, Freiburg, Germany; ^7^ Department of Neuroradiology, Medical Center - University of Freiburg, Faculty of Medicine, University of Freiburg, Freiburg, Germany; ^8^ Center for Basics in Neuromodulation, Faculty of Medicine, University of Freiburg, Freiburg, Germany; ^9^ Department of Nuclear Medicine, Medical Center - University of Freiburg, Faculty of Medicine, University of Freiburg, Freiburg, Germany; ^10^ Department of Neurology and Experimental Neurology, Charité - Universitätsmedizin Berlin, Berlin, Germany; ^11^ German Center for Neurodegenerative Diseases (DZNE) Berlin, Berlin, Germany

**Keywords:** autoimmune encephalitis, encephalopathy, autoimmune psychosis, antibodies, schizophrenia, catatonia

## Abstract

**Background:**

Autoimmune encephalitis, such as anti-NMDA-receptor encephalitis, typically presenting with subacute onset of neuropsychiatric symptoms, can be detected by antineuronal autoantibodies or inflammatory changes in the cerebrospinal fluid (CSF), as well as pathological alterations in electroencephalography (EEG), magnetic resonance imaging (MRI), or [18F]fluorodeoxyglucose positron emission tomography (FDG PET). For patients with predominant psychotic symptoms, the term autoimmune psychosis was proposed. Here, the authors present the case of a patient with probable autoimmune psychosis associated with unknown antineuronal antibodies.

**Case Presentation:**

A 18-year-old male patient with preexisting autism spectrum disorder developed a severe catatonic syndrome over 2.5 years. The MRI showed normal findings, the EEG depicted intermittent slowing, and the independent component analyses showed additional sharp spikes. However, FDG PET, the basic laboratory analysis and testing of the serum/CSF for well-characterized antineuronal autoantibodies were unsuspicious. The serum and CSF “tissue-based assay” using indirect immunofluorescence on unfixed murine brain tissue revealed antineuronal autoantibodies against an unknown epitope in granule cells in the cerebellum and to neurites of hippocampal interneurons with a somatodendritic staining pattern. The immunosuppressive treatment with high-dose glucocorticoids, plasma exchange, and rituximab led to partial improvement.

**Conclusion:**

The patient probably suffered from autoantibody-associated autoimmune psychosis. The special features of the case were that the patient (1) presented with mostly inconspicuous basic diagnostics, except for the altered EEG in combination with the detection of CSF autoantibodies directed against a currently unknown epitope, (2) experienced an isolated and long-lasting psychotic course, and (3) had pre-existing autism spectrum disorder. The detection of a probable autoimmune pathophysiology in such cases seems important, as it offers new and more causal immunosuppressive treatment alternatives.

## Background

Autoimmune encephalitis, such as anti-NMDA-receptor encephalitis, is typically associated with a subacute onset of neuropsychiatric symptoms, and can often be identified by the presence of antineuronal autoantibodies or inflammatory changes in the cerebrospinal fluid (CSF), electroencephalography (EEG) pathologies, and evidence of encephalitis in magnetic resonance imaging (MRI) or [18F]fluorodeoxyglucose positron emission tomography (FDG PET; 1–5). Until now, a series of antineuronal autoantibodies against surface (e.g., NMDA-R, LGI1, CASPR2, AMPA1/2-R, GABA-B-R, DPPX) or intracellular antigens (e.g., Yo, Hu, CV2/CRMP5, Ri, Ma1/2, SOX1, GAD65, amphiphysin), or “potentially antineuronal” systemic antibodies (e.g., antinuclear antibodies [ANAs] against double-stranded [ds]-DNA or gliadin autoantibodies) (3–5) are known. The associated neuropsychiatric syndromes are classically characterized by a combination of neurological (e.g., seizures, movement disorders, focal-neurological deficits, or reduced consciousness) and psychiatric (e.g., psychosis, mania, or catatonia) symptoms (3, 6, 7). However, there are also variants with milder isolated or predominant psychiatric symptoms described in the literature associated with some of the autoantibodies (8–11). Catatonic syndromes are typically reported in anti-NMDA-receptor encephalitis (12). Some authors have suggested the term “autoimmune psychosis” to describe that subgroup of patients with predominant psychotic symptoms (13–15). New methods (e.g., “tissue-based assays” *via* indirect immunofluorescence on unfixed murine brain tissue) can be used to increase sensitivity in discovering also novel autoantibodies against so far unknown epitopes on the cell surface (16). Previously “seronegative” autoimmune neurological syndromes have already been confirmed by such positive tests (17). The role of tissue-based assays in the detection of autoimmune psychoses is largely unclear. The rationale of the current case report is to present a patient with probable autoimmune psychosis associated with antineuronal autoantibodies directed against a currently unknown antigen detected by a tissue-based assay.

## Case Presentation

Here, the authors present the case of a 18-year-old male German pupil who developed a severe catatonic syndrome over 2.5 years. The catatonic syndrome presented with symptoms of mutism (the patient did not speak at all during the course of the disease), catalepsy and rigor, echo phenomena, and intermittent states of psychomotor agitation (the patient would run up and down the hallway in an excited manner). Further, he developed fecal and urinary incontinence, displayed impaired perception and formal thought disorder, exhibited severe concentration and attention deficits, and reported delusions (e.g., he thought he was telepathically controlled) and auditory hallucinations hearing commenting voices (e.g., he heard songs and the voice of a classmate that would tell him funny things). The symptoms initially began with cognitive deficits and seizure-like states with laughing. An epileptic examination by telemetry did not reveal any evidence of gelastic seizures. Auditory hallucinations probably existed for about 1.5 years. In addition, the patient developed an increased muscle tone and cramps of the tongue for half a year. These were interpreted as tardive dyskinesias after risperidone intake. At that time, due to severe abulia and akinetic mutism he had to be fed *via* stomach tube and in retrospect probably also suffered from headache.

### Diagnostic Findings

**Figure 1:**
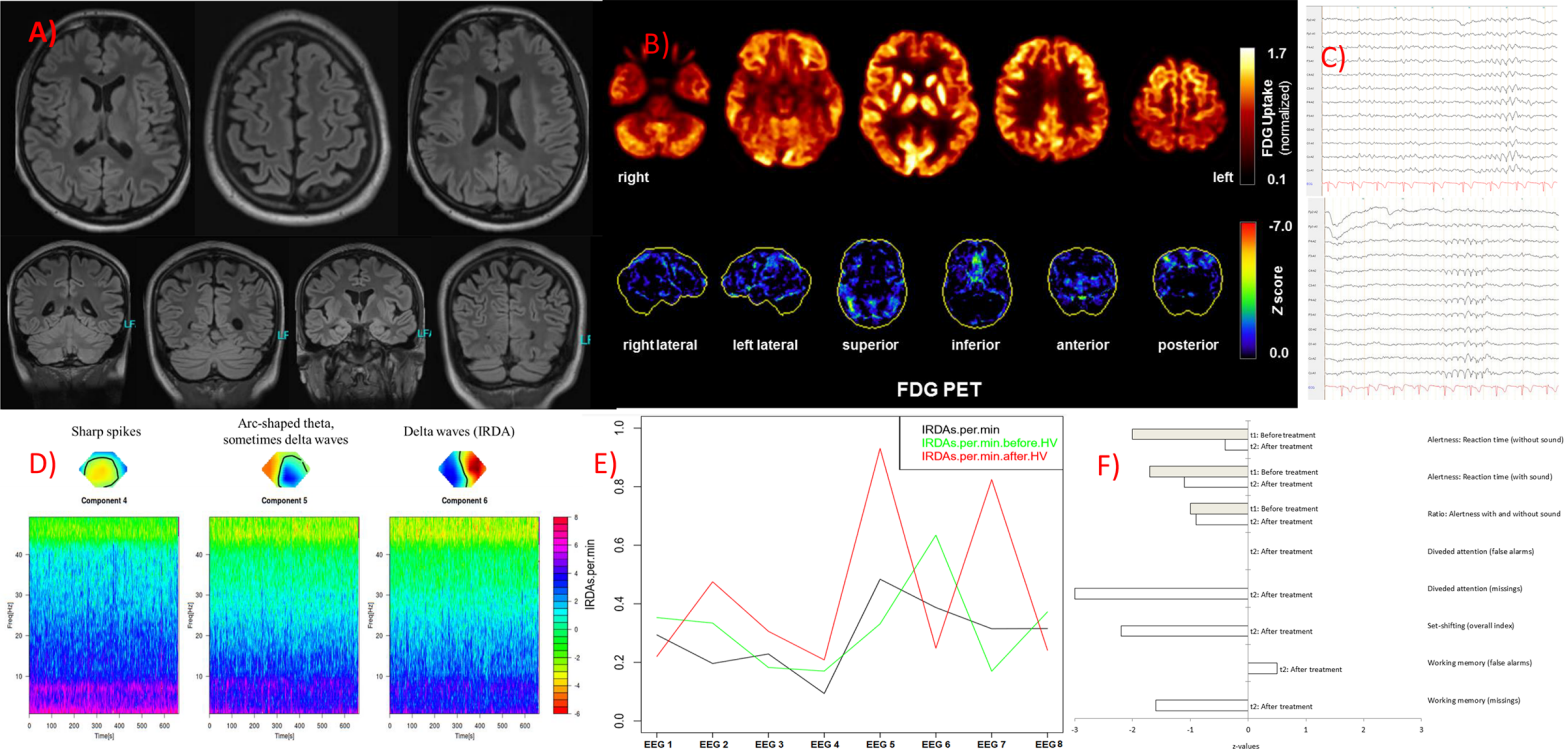
Imaging, electroencephalography (EEG) and neuropsychological findings. **(A)** The magnetic resonance imaging produced unsuspicious findings. **(B)** An [^18^F]fluorodeoxyglucose positron emission tomography (FDG PET) examination at baseline revealed no relevant regional metabolic abnormality suggestive of inflammation. Upper row: Transaxial FDG PET images (voxel-wise FDG uptake normalized to whole brain uptake), lower row: 3D surface projections of regions with decreased FDG uptake (colour-coded Z-score, compared to age-matched healthy controls; minor, linear-shaped areas of hypometabolism were judged to be non-specific partial volume effects due to atrophy; 18). The cerebral FDG PET scan was performed as part of the whole-body PET/CT scan 50 min after injection of 340 MBq FDG (Vereos Digital PET/CT, Philips Healthcare, The Netherlands). **(C)** The routine EEG revealed marginal intermittent, rhythmic, and generalized slowing. **(D)** The independent component analyses showed sharp spikes in component 4, arc-shaped theta (sometimes delta waves) in component 5, and intermittent rhythmic, generalized delta activity (IRDAs) in component 6. **(E)** Rate of IRDAs during initial treatment: Under benzodiazepines (EEG 1, 3 and 4) the IRDA activity appeared to be low, after the steroid pulse (5) the IRDA rate increased significantly, during plasma exchange (6) they were reduced again, in the two follow-up EEGs directly after initial treatment (7) and half a year later under rituximab (8) the IRDA activity decreased overall, whereas without valproate the IRDAs were increased again after hyperventilation (7). EEG 1 was performed with a medication of lorazepam, amisulpride, and olanzapine; EEG 2 with valproate (lamotrigine in low dose) and amisulpride; EEG 3 with clobazam, valproate (lamotrigine in low dose), and amisulpride; EEG 4 with clobazam (low dose), valproate (lamotrigine in low dose), and amisulpride; EEG 5 with valproate (lamotrigine in low dose), amisulpride; after steroid pulse treatment, with still 40 mg methylprednisolone orally; EEG 6 with valproate (reduced), lamotrigine, amisulpride, still 10 mg methylprednisolone and after 4 cycles of plasma exchange; EEG 7 with valproate (low dose), lamotrigine, amisulpride, with still 10 mg methylprednisolone orally; EEG 8 follow up (a half year later) with lamotrigine and amisulpride. **(F)** Measures of attentional performance during the course of the disease (t2 was performed at six-months follow-up).

**Figure 2:**
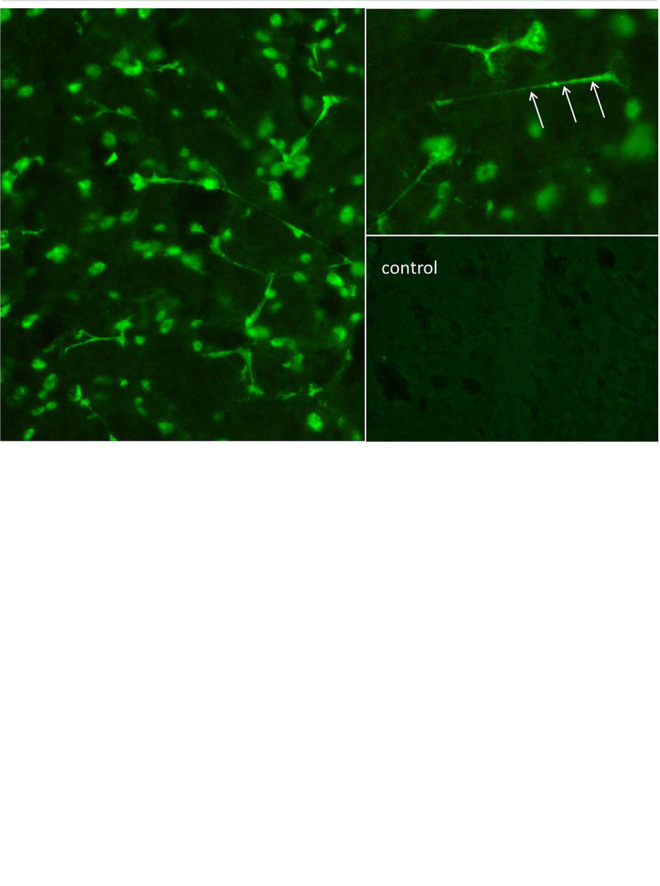
The “tissue-based assay” using indirect immunofluorescence screening on unfixed brain sections of rodents identified antibody binding to granule cells in the granule cell layer of the cerebellum (above) and to neurites of hippocampal interneurons (below). Autoantibodies in the cerebrospinal fluid showed a “somatodendritic staining pattern” (arrows point to dendritic processes on hippocampal interneurons in stratum lacunosum moleculare), but also to cell nuclei, reflecting antinuclear antibodies (ANAs). In a control cerebrospinal fluid sample of the hippocampus (downright) there was no comparable signal. The cerebrospinal fluid material was used undiluted. ML, molecular layer; GCL, granule cell layer; WM, white matter.

The diagnostic examinations were carried out at first presentation in our tertiary care hospital, approximately 2.5 years after symptom onset. The structural brain MRI was normal (**Figure 1**). There were no white matter or contrast-enhanced lesions. A routine EEG depicted intermittent, generalized, but frontally accentuated slowing. The independent component analyses (ICA) revealed sharp waves (component 4) and intermittent rhythmic delta activity (IRDA, component 5 + 6, **Figure 1**). In the serum, increased ANA titers (1:800) without specificity against a defined cluster of extractable nuclear antigens (ENA) including double-stranded (ds)-DNA were found. The patient’s basic CSF analytic results (white blood cell count, albumin quotient, IgG-index, and oligoclonal bands) were normal. No antibodies against the intracellular (synaptic) antigens Yo, Hu, CV2/CRMP5, Ri, Ma1/2, Tr, Zic4, GAD65, and amphiphysin were found. Initially, anti-Sox1 autoantibodies were once weakly positive, in the course no more. Antibodies against various well-characterized neuronal cell surface antigens (NMDA-R, LGI1, CASPR2, AMPA1/2-R, GABA-B-R, DPPX) were also negative. However, in his serum and CSF, autoantibody binding to granule cells in the granule cell layer of the cerebellum and to neurites of hippocampal interneurons with a somatodendritic staining pattern against an unknown neuronal epitope were found in addition to an ANA pattern (**Figure 2**). An FDG PET examination of his brain showed no relevant regional hypermetabolism (suggestive of active inflammation) or hypometabolism (as a possible sequel of inflammation or degeneration) aside from mild relative striatal hypermetabolism, compatible with ongoing neuroleptic medication (**Figure 1**). A whole-body FDG PET/computer tomography (CT) detected no metabolic or structural pathologies suggestive of malignancy or inflammation. All diagnostic investigations and findings are summarized in **Table 1**.

**Table 1:** Diagnostic findings (approximately 2.5 years after symptom onset).

**Neuropsychiatric and general medical examination**	Psychiatric/neurological: Severe formal thought disorder and attention as well as concentration deﬁcits, parathyme flattened mood. Changing energy level. Abulia, loss of interests, dramatically reduced activity, akinetic mutism. Delusions/auditory hallucinations, no suicidal tendencies, no sleep disorders, normal appetite. Catalepsy and increased muscle tone, echo phenomena and intermittent excitation states. No focal neurological signs. Internal: Fecal and urinary incontinence.
**Blood analyses**	Blood cell count, electrolytes, liver/kidney/pancreas values, and C-reactive protein were normal. Folic acid was normal. Vitamin B12 was high (887 pg/ml; reference: 771 pg/ml), and selenium was decreased (60; reference: 75-140 µg/l). Vitamin D was suboptimal (21 ng/ml; optimal: >30 ng/ml). Thyroid-stimulating hormone, triiodothyronine, and thyroxine levels were in normal ranges. Autoantibodies against thyroglobulin, TSH receptor and thyroid peroxidase were not detectable. Antibody testing for Lyme borreliosis, syphilis and HIV were negative. No IgG antibodies against the intracellular onconeural antigens Yo, Hu, CV2/CRMP5, Ri, Ma1/2, SOX1, Tr, Zic4 or the intracellular synaptic antigens GAD65/amphiphysin were found (using Ravo line assay^®^). Sox1 IgG autoantibodies were once weakly positive, in the course no more. IgG antibodies against different neuronal cell surface antigens (NMDA-R, AMPA-1/2-R, GABA-B-R, DPPX, LGI1, CASPR2) were negative (using Euroimmun biochip-assays^®^). Aquaporin 4 and MOG antibodies were negative. Positive “tissue-based assay” for unknown antineuronal antibodies with somatodendritic staining pattern binding to granule cells in the granule cell layer of the cerebellum and to neurites of hippocampal interneurons. Screening for serum antinuclear antibodies (ANA) using indirect immunofluorescence (IIF) on HEp-2000^®^ cells (Immuno Concepts, Sacramento, CA, USA) showed increased titers (1:800; reference < 1:50) without specificity against extractable nuclear antigens (ENA, lineblot assay including nRNP/Sm, Sm, SS-A, Ro-52, SS-B, Scl-70, PM-Scl,Jo-1, CENP-B, PCNA, dsDNA, nucleosomes, histones, ribosomal-P-proteins, AMA-M2, and DFS70 (ANA-Profile 3 plus DFS70, Euroimmun, Luebeck, Germany) or double-stranded (ds)-DNA (IgG-ELISA, Euro-Diagnostica, Malmö, Sweden). Anti-neutrophil cytoplasmic antibodies (ANCA), antiphospholipid antibodies, and rheumatoid factor were negative. Anti-mitochondrial (AMA) and anti-smooth muscle antibodies (SMA) were borderline positive (+). Analyses of the complement system (C3, C4, CH50 and C3d) showed no relevant findings. Normal serum IgA, IgM und IgG immunoglobulin concentrations; immunofixation showed no monoclonal antibody production. Lymphocyte immunophenotyping by fluorescence-activated cell sorting (FACS) analysis showed only a slightly decreased percentage of total lymphocytes (24.7%; reference 35-45%) but no relevant changes in lymphocyte subsets.
**Cerebrospinal fluid analyses**	Normal white blood cell count (1/µL; reference <5/µL). Normal protein concentration (349 mg/L; reference <450 mg/L), and normal age-corrected albumin quotient: 3.4; age-dependent reference < 6.5 × 10^–3^). No CSF specific oligoclonal bands; IgG index not increased (0.6; reference ≤0.7). CSF lactate not increased (1.51 mmol/l; reference 1.5-2.1 mmol/L). No IgG antibodies against the intracellular onconeural antigens Yo, Hu, CV2/CRMP5, Ri, Ma1/2, SOX1, Tr, Zic4 or the intracellular synaptic antigens GAD65/amphiphysin were found (using Ravo line assay^®^). IgG antibodies against neuronal cell surface antigens (NMDA-R, AMPA-1/2-R, GABA-B-R, DPPX, LGI1, CASPR2) were negative (Euroimmun Biochip assay^®^). Positive “tissue-based assay” for unknown antineuronal antibodies with somatodendritic staining pattern binding to granule cells in the granule cell layer of the cerebellum and to neurites of hippocampal interneurons.
**Cerebral magnetic resonance imaging**	Basically inconspicuous, except for a discreet and query atrophy that was judged to be insignificant by neuroradiologists, and pineal cyst (with maximum sagittal diameter of up to 11.5 mm).
**Electro-encephalography**	Intermittently generalized, rhythmic, frontally accentuated slow wave activity. The independent component analyses showed sharp spikes in component 4, arc-shaped theta (sometimes delta waves) in component 5, and delta waves (IRDAs) in component 6 (**Figure 1**).
**[^18^F]fluorodeoxy-glucose positron emission tomography (FDG PET)**	Unsuspicious brain metabolism. No lesions/metabolic changes suspicious of malignancy on whole-body FDG PET/computer tomography.
**Heart examination**	Inconspicuous resting electrocardiography.

### Illness, Somatic, and Family Histories

Past medical history was negative for in-utero or birth complications, febrile convulsions or epileptic seizures, inflammatory brain diseases, relevant systemic infections, or craniocerebral traumata with unconsciousness. Since the first decade, he suffered from autism spectrum disorder with difficulties in social cognition, communication and interaction with peers like playing with other kids, ritualized repetition of everyday situations/dialogues, and special interests for automatic machines and trains. He had no history of autoimmune disease, infection, cancer, or other relevant somatic disorders. His family history (for parents and grandparents, he has no siblings) was devoid of any diagnosed psychiatric disorders. His mother suffers from psoriasis. The paternal grandfather died of a glioblastoma, the maternal grandfather had an unclear tumor disease of the eye.

### Treatment and Outcome

The classical neuroleptic treatment with risperidone, olanzapine, and amisulpride, as well as treatment with lorazepam, did not lead to relevant improvement. An increase in extrapyramidal motor symptoms was observed with both risperidone and amisulpride application in a dose-related extent. Under valproate treatment, the mutism improved slightly (so that basic communication became possible). Of the three different IRDA components visible before valproate (**Figure 1**), only one single left frontal component was still visible in the ICA after valproate. Due to an increase in weight as a probable side effect of valproate, the medication was changed to lamotrigine. Because of the refractory disease course and the suspected autoimmune origin, the diagnosis of a probable autoimmune psychosis was made, and immunosuppressive treatment was initiated. Treatment consisted of high-dose glucocorticoid pulse therapy (1,000 mg methylprednisolone daily for five days), followed by oral treatment and stepwise glucocorticoid tapering. Plasma exchange (eight cycles) was used to rapidly decrease autoantibody concentrations. For maintenance treatment the B-cell depleting anti-CD20 antibody rituximab was given intravenously in a dose of 2x1,000 mg within 14 days and subsequently 1,000 mg every six months. The treatment steps were carried out in succession. Following high-dose glucocorticoid treatment and plasma exchange, slight improvements were observed. His incontinence improved and he made considerable progress in his ability to communicate. At the first six-month follow-up (the admission was made for the third rituximab treatment), the patient had gone back to school to gain structure in his daily routine (initially 3 h, later the whole day). A complete neuropsychological testing was possible for the first-time half a year after initiation of immunosuppressive treatment, showing ongoing deficits in alertness and working memory and particularly in divided attention and set shifting (**Figure 1**, t2). His formal thinking and cognitive functions were still compromised; intermittently there were long response latencies. Auditory hallucinations remained, however, on a reduced level, delusions did not occur any more. In summary, there was a relevant clinical improvement, but the patient was still significantly limited in his ability to function in everyday life. The follow-up MRI was unchanged, and CSF autoantibody testing showed reduced titers of the anti-neuronal autoantibody, although still clearly detectable. **Figure 1** illustrates the course of the EEG slowing (IRDAs) under treatment and after a 6-months follow-up period.

## Discussion

The present case report describes a male patient suffering from a probable autoantibody-associated psychotic syndrome presenting with severe catatonic and paranoid-hallucinatory symptoms. Striking aspects of the presented case are: (1) the mostly non-specific basic diagnostics in combination with the detection of antineuronal autoantibodies showing a “somatodendritic staining pattern” against an unknown neuronal epitope, (2) the long-lasting psychiatric course, (3) the slight improvement under anticonvulsants and clear improvement under immunosuppressive treatment, and (4) the preexisting autism spectrum disorder.

### Diagnostic Considerations

The initial comprehensive diagnostic procedure revealed no clear pathological findings indicating an immunological pathophysiology. The basic CSF diagnostics and the biochip assay tests on fixed cells for detecting established antineuronal autoantibodies, the MRI of the brain, and the FDG PET failed to identify any relevant abnormality. The EEG showed non-specific changes in the form of an intermittent frontally accentuated slowing. The ICA helped to demonstrate electrophysiological instability with sharp spikes and IRDAs. Even with this comprehensive diagnostic procedure, the identification of an autoimmune psychosis would not have been possible. In the presented case, the critical clues were only obtained from a tissue-based assay *via* indirect immunofluorescence on unfixed murine brain tissue carried out in a specialized CSF laboratory. Antineuronal autoantibody binding to granule cells in the granule cell layer of the cerebellum and to neurites of hippocampal interneurons against an unknown epitope was found. The immunofluorescence pattern was a “somatodendritic staining pattern”, increasing the likelihood of functional relevance of the autoantibody (19). If or not the absence of encephalitis signs in the CSF or FDG PET are related to the long development or the isolated psychiatric course of the disease remains unclear. For some other well-characterized antineuronal antibodies such as LGI1 or IgLON5, an inconspicuous CSF is also common. However, based on present conceptualization, this case can most likely be taken as an example of a probable autoimmune psychosis compatible with a “mild encephalitis” concept (20), even though it would not fulfill the current consensus criteria of probable autoimmune encephalitis (3).

### Special Clinical Characteristics

From a clinical point of view, it was interesting to observe that the catatonic symptoms and the EEG pathologies already improved slightly following initial treatment with valproate. Patients with confirmed autoimmune encephalitis (e.g., anti-NMDA-R encephalitis) also often improve initially under “symptomatic” treatment with neuroleptics. The response to psychotropic drugs such as neuroleptics or, in this case, anticonvulsants, is therefore not a contradiction to an autoimmune genesis. For the presented case, it can be speculated that neuronal network instability caused by the detected unspecified autoantibodies improved under anticonvulsive (i.e., network-stabilizing) medication (see **Figure 1**). This idea would be well explained by the Local Area Network Inhibition (LANI) hypothesis. Following the LANI model, the excitatory spikes and IRDAs could lead to a counterregulatory hyperinhibition of neuronal networks and associated symptoms (e.g., cognitive dysfunction; 21). Finally, in our case report, the link to autism is interesting. In their clinical work, the authors of this paper repeatedly observed patients with developmental disorders who later developed autoimmune encephalitis. It would be interesting to analyze whether autism spectrum diseases with their well-known link to immunological abnormalities (e.g., autoantibodies against brain tissue, microglia activation, inflammatory cytokines; 22) are associated with an increased risk for the subsequent development of autoimmune encephalitis. In the present case, an immunological predisposition with increased titers of antinuclear antibodies in a male patient and a positive family history for immunological disorders (mother suffers from psoriasis) was present. Initial studies in children even showed that a small subgroup of children with autism displayed evidence of autoimmune encephalitis (23).

## Limitations

A pathophysiological significance of the serum/CSF autoantibodies is probable due to the antineuronal pattern in combination with intermittent EEG slowing and sharp spikes. The response to immunosuppressants was only partial and without full remission of symptoms; this could be due to the autoantibody itself or to the long course of the disease. Precise titer determination of the detected antineuronal antibodies and confirmation tests with immunohistochemistry on hippocampal neurons were not performed in the presenting patient; in similar cases in the future, the antibody findings should be analyzed in more detail (see 24). Also, CSF may be subjected to single-cell antibody repertoire analysis, which allows identification of disease-related monoclonal human autoantibodies and their targets, and seems to become the gold-standard also for determining pathogenicity of neuropsychiatric autoantibodies (25, 26). A better identification of the target epitope would help to identify the underlying pathophysiological processes more clearly. Another limiting factor in the current case is that the anti-GABA-A-R antibodies also were not analyzed using a cell-based assay. However, the lack of laminar neuropil binding in the tissue-based assay, as would be seen in autoimmune encephalitis with anti-GABA-A-R antibodies (or anti-NMDA-R or anti-GABA-B-R antibodies), argues against the presence of these antibodies. Larger patient numbers in future studies are required to determine if the hypothetical clinical characteristics discussed here with regard to the LANI hypothesis and the association with autism are correct.

## Conclusions

This case report shows an association of a psychotic syndrome with predominant catatonic symptoms and antineuronal autoantibodies against an unknown epitope detected by a tissue-based assay. The basic diagnostics were mostly unremarkable with the exception of a conspicuous EEG. The application of tissue-based assays for the detection of so far unknown autoantibodies might also be helpful in other psychiatric patients with suspected autoimmune pathophysiology.

## Data Availability Statement

All datasets necessary for this case study are included in the article.

## Ethics Statement

The patient and his parents have given their signed written informed consent for this case report, including the presented images, to be published.

## Author Contributions

DE, PS, SR, AP, and LT treated the patient. DE performed the data research and wrote the paper. SR, AP, and HP performed the neurological interpretation. SR performed the CSF basic analyses. HP performed the tissue-based testing on unfixed brain sections. BF and LT performed the EEG analyses. NV performed the rheumatological tests and immunological interpretation. TS performed the neuropsychological testing and interpretation. PM performed the nuclear medicine investigations and interpretation. KE, KN, and SM performed and interpreted the MRIs. AP performed the rituximab treatment. KR, BF, DD, and KD supported the clinical and laboratory interpretation. All authors were critically involved in the theoretical discussion and composition of the manuscript. All authors contributed to the article and approved the submitted version.

## Funding

The article processing charge was funded by the German Research Foundation (DFG) and the University of Freiburg in the funding program Open Access Publishing.

## Conflict of Interest

SR: Receiving consulting and lecture fees, grant and research support from Bayer Vital, Biogen, Merck Serono, Novartis, Sanofi-Aventis, Genzyme, Roche and Teva. Furthermore, SR indicates that he is a founding executive board member of ravo Diagnostika GmbH Freiburg. NV: Advisory boards, lectures and travel grants from Roche. KD: Steering Committee Neurosciences, Janssen. PM: Advisory boards and lectures within the last three years: GE, Philips, OPASCA, Curium, Desitin and Medac. LT: Advisory boards, lectures, or travel grants within the last three years: Roche, Eli Lilly, Janssen-Cilag, Novartis, Shire, UCB, GSK, Servier, Janssen and Cyberonics.

The remaining authors declare that the research was conducted in the absence of any commercial or financial relationships that could be construed as a potential conflict of interest.

## Abbreviations

ANA, antinuclear antibody; CSF, cerebrospinal fluid; CT, computer tomography; ds, double-stranded; EEG, electroencephalography; ENA, extractable nuclear antigens; FDG-PET, [18F]fluorodeoxyglucose positron emission tomography; ICA, independent component analyses; IRDA, intermittent rhythmic delta activity; LANI, Local Area Network Inhibition; MRI, magnetic resonance imaging.
